# Lactate as a Preoperative Predictor of Mortality in Patients Undergoing Emergency Type A Aortic Dissection Repair

**DOI:** 10.3390/jpm15050211

**Published:** 2025-05-21

**Authors:** Sombuddha Bhadra, Rachel H. Drgastin, Howard K. Song, Frederick A. Tibayan, Gurion Lantz, Julie W. Doberne, Castigliano M. Bhamidipati

**Affiliations:** Division of Cardiothoracic Surgery, Department of Surgery, Oregon Health & Science University, 3181 SW Sam Jackson Park Road, MC #L353, Portland, ON 97239, USA; bhad1000@pacificu.edu (S.B.); drgastin@ohsu.edu (R.H.D.); songh@ohsu.edu (H.K.S.); tibayan@ohsu.edu (F.A.T.); lantz@ohsu.edu (G.L.); doberne@ohsu.edu (J.W.D.)

**Keywords:** aortic dissection, Stanford Type A aortic dissection, lactate dehydrogenase, lactate, malperfusion, mortality

## Abstract

**Background:** Aortic dissection is a life-threatening condition where emergent surgical repair is the standard of care. However, despite operative intervention, mortality is 10–15% in all patients. Objective markers to distinguish when surgical repair is more beneficial versus being futile are warranted. Currently, no such known measures are widely agreed upon. Since most complications from aortic dissection stem from malperfusion, serum lactate is thought to be a surrogate marker for malperfusion. This scoping review aims to examine the preoperative predictive value of lactate or lactate dehydrogenase (LDH) in assessing postoperative mortality in patients undergoing surgical repair for acute Stanford Type A aortic dissection (ATAAD). **Methods:** PubMed was searched for the following search terms: “Dissection, Ascending Aorta”, “Dissection, Thoracic Aorta”, or “Aortic Dissection”. Prospective and retrospective randomized controlled trials, case reports, and cohort studies were included in the initial search. Studies were first screened for inclusion of preoperative lactate or LDH level with a search of “lac” or “LDH”. Included studies consisted of patients aged 18 or older diagnosed with Stanford Type A/Debakey Type I and II aortic dissection with reported preoperative lactate or LDH levels and postoperative mortality treated within 14 days of symptom onset. Preoperative laboratory values were measured from samples collected prior to patient transfer to the operating room or before utilization of ECMO intraoperatively. **Results:** A comprehensive database search identified a total of 4722 articles. After a rigid screening process, 46 studies fit the inclusion criteria. These papers reported a combined 4696 participants with either preoperative lactate or LDH levels and postoperative mortality. The mean preoperative lactate level was 2.4 mmol/L, whereas the LDH level was 424.9 U/L. Postoperative mortality was 16.51%. Average creatinine, BUN, platelets, INR, PT, PTT, and hemoglobin were all within normal lab analysis limits. **Conclusions:** Neither lactate nor LDH should be used as a solo predictor of postoperative mortality after ATAAD due to lack of consensus on the cut-off values. Accompanying clinical signs, lab abnormalities, and radiographic findings taken together may be better predictors of prognosis.

## 1. Introduction

Acute Type A aortic dissection (ATAAD) is life-threatening and, without intervention, leads to mortality in >58% of unoperated patients [1,2]. Under the recent Society for Vascular Surgery (SVS) and Society of Thoracic Surgeons (STS) classification scheme for aortic dissection, ATAAD represents dissection in which the entry tear originates in the proximal ascending aorta, or zone 0 [3,4]. Emergency surgery is associated with 12% to 47% mortality [2,5,6,7,8]. Despite high postoperative mortality rates, many ATAAD repairs are completed with preoperative indicators of unsuccessful outcome (i.e., malperfusion, high lactate, high LDH, debilitating cerebrovascular impairment, and extended time from initial ATAAD to restored antegrade aortic flow).

Personalized medicine aims to tailor medical and surgical care to each individual patient, incorporating biomarkers and predictive tools to optimize outcomes and expectations. In the context of ATAAD, where patient presentations vary widely, individualized assessments refine care planning and decision-making [9]. However, there is no consensus on whether patients could benefit from having malperfusion addressed before undergoing surgical repair. Recently, a calculator developed from the German Registry for ATAAD (GERAADA) has been created and externally validated to predict the 30-day mortality rate for patients undergoing ATAAD operative repair [10,11,12]. Sequelae of ATAAD are based on preoperative indicators of poor outcomes, including shock at presentation, cardiac tamponade, myocardial infarction, reduced ejection fraction, neurologic changes, severe aortic regurgitation, cardiopulmonary resuscitation, renal failure, and, most notably, local and systemic malperfusion [5,13,14,15]. A simple and surrogate marker of malperfusion could help risk stratify patients expected to have a poor outcome, especially when calculators or other sophisticated tools are not utilized. Serum lactate is a frequently used marker to aid in assessing tissue perfusion in distributive shock. Separately, ATAAD causes malperfusion and tissue hypoxia, leading to profound shock, and this needs further exploration [16]. Measuring preoperative serum lactate levels that examine end-organ transition to the lactic acid fermentation pathway in the setting of tissue malperfusion can be instructive [17].

Although studies have investigated using lactate as an independent predictor of mortality, there is no consensus threshold of preoperative lactate above which there may be minimal potential benefit from surgical repair [5,18,19]. There is also a lack of clarity on the protocol for alternatives in the circumstance that a patient’s lactate is too high—for example, when does regional malperfusion management first make more sense over central ATAAD repair? This scoping review aims to summarize and interpret the literature by considering relevant studies with reported preoperative lactate or LDH values and postoperative mortality to inform whether the lactate pathway can be used as an independent predictor of mortality or if lactate levels should be used in conjunction with other corroborative data.

## 2. Methods

**Figure 1:**
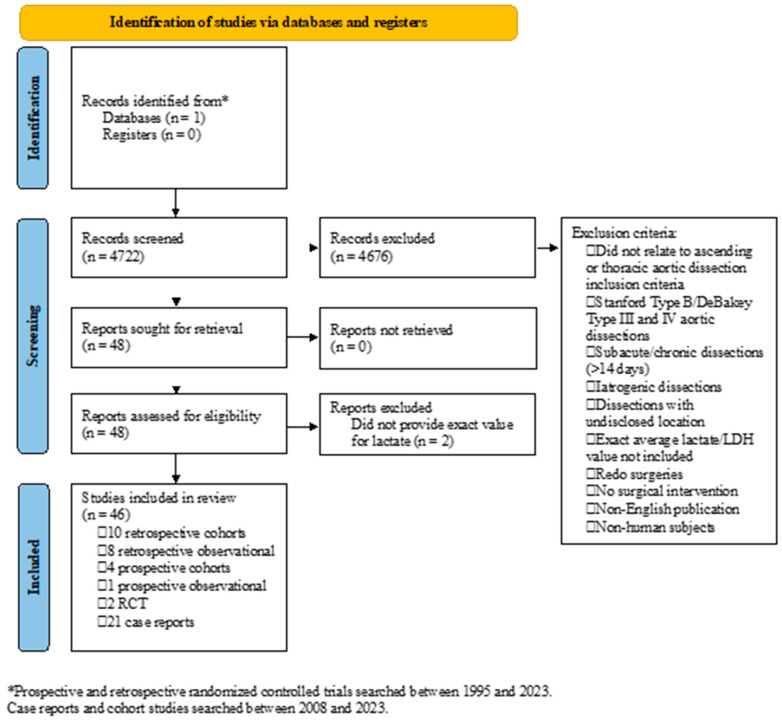
Identification of studies.

PubMed was searched, and studies were included for screening if they specifically returned manuscripts after the following search terms: “Dissection, Ascending Aorta”, “Dissection, Thoracic Aorta”, or “Aortic Dissection”; lactate-specific search terms were not included to reduce bias and risk of elimination. Prospective and retrospective randomized controlled trials (RCTs) between 1995 and 2023 and case reports plus cohort studies between 2008 and 2023 were included in the initial search. The latter only included the past 15 years due to the clinical relevance of more contemporary repairs (*n* = 3558) and were included to provide insights into rare but clinically relevant presentations. A single independent reviewer (SB) iteratively screened the abstracts, and no automation tools were used. Studies were first screened for inclusion of preoperative lactate or LDH level with a search of “lac” or “LDH”. Studies were included from adult patients (>18 y) diagnosed with Stanford Type A/Debakey Type I and II aortic dissection where preoperative lactate or LDH levels were reported and where postoperative mortality within 14 days of symptom onset or operation was reported. Preoperative laboratory values from samples intraoperatively collected prior to patient transfer to the operating room or before utilization of ECMO were included. Studies were excluded if they did not relate to ascending or thoracic aortic dissection, included patients with Stanford Type B/Debakey Type III and IV aortic dissections, subacute/chronic dissections (>14 days), iatrogenic dissections, or dissections with undisclosed location, did not specify an exact average lactate/LDH value for all participants, included redo surgeries, included patients who chose comfort care, were published in a language other than English, or included non-human subjects (Figure 1). Mean values were used to interpret the data trends due to the heterogeneity of study designs, and since this review focused on broad aspects, a systematic review or meta-analysis was not feasible. This study did not involve human subjects and was conducted using publicly available data sources; therefore, IRB approval/informed consent was not required.

## 3. Results

A comprehensive search using the terms mentioned above resulted in 4722 articles. Following the screening process and after the inclusion and exclusion criteria were applied (Figure 1), 46 studies were included for this review. The studies included ten retrospective cohort studies, eight retrospective observational studies, four prospective cohort studies, one prospective observational study, two randomized controlled trials, and twenty-one case reports. In total, data on 4696 participants were reported. Other than case reports, which reported on one patient each, sample sizes varied from 10 to 632 patients. The mean age ranged from 47 y to 69 y, while the proportion of males ranged from 15.8% to 95.2%. The age of patients in case reports ranged from 31 y to 83 y, and 16 of 21 case reports reported data from male patients. Preoperative lactate was reported in 42 studies, while LDH was reported in the remaining 4 studies. The postoperative mortality period ranged from the index in-hospital to 1 y following surgery. Lactate or LDH values and postoperative mortality were reported in all studies included in this analysis. BUN was reported in five studies, creatinine in twenty-four manuscripts, INR in three reports, PT and PTT in two studies, platelets in eight studies, and hemoglobin in eleven manuscripts. The demographics, mean preoperative lab findings, and postoperative mortality are shown in Table 1.

Of the 4695 participants, 1567 were women (33.4%). Mean preoperative lactate and LDH were the only variables that were found to be elevated compared to their normal values at 2.4 mmol/L and 424.9 U/L, respectively. Mean creatinine (1.06 mg/dL), INR (1.2), PT (13.67 s), PTT (35.38 s), platelets (161,000 platelets/µL), and hemoglobin (12.57 g/dL) were all within normal limits. Overall, postoperative mortality, taken together from in-hospital to 1 y post-op after ATAAD, was 16.8% (Table 1). Patients with lactate levels ≥3.0 mmol/L showed an increased average mortality rate compared to those below this threshold (21.4%, *n* = 899; 15.9%, *n* = 3744). However, the optimal cutoff value remains unclear, and the ≥3.0 mmol/L cutoff value was only used for data interpretation.

## 4. Discussion

The authors thoroughly reviewed the current literature evaluating the role of preoperative lactate levels in determining prognosis after surgical repair. Due to the high risk of morbidity and mortality associated with acute Type A aortic dissection (ATAAD) even with surgical repair, determining prognosis using preoperative markers can help physicians assess the benefit or futility of a procedure. Serum lab values are one of the easiest and most widely available indicators of the severity of disease. Specifically, serum lactate is a simple and swiftly obtained surrogate for malperfusion and can be helpful in risk stratification of emergent ATAAD. Of the preoperative lab values examined in this analysis, only lactate and LDH levels were found to be elevated at 2.4 mmol/L and 424.9 U/L, respectively. BUN, creatinine, INR, PT, PTT, platelets, and hemoglobin were all within normal limits. These data add to the growing literature that correlates elevated lactate values with poorer outcomes in ATAAD and should be included in the workup when the diagnosis is suspected. In fact, Ghoreishi et al. [21] suggested using lab markers such as lactate, creatinine, and liver function tests and has been externally validated [22]. However, given the lack of consensus on optimal cut-off value, lactate should not be used as an independent predictor of outcomes. Rather, it should be an integral part of a criterion using other easily obtainable data points such that it is practical and implementable in an emergent situation. To the authors’ best knowledge, there is no consensus research outlining a protocol to delay surgical intervention in the case of high lactate levels. Due to the lack of widely acceptable criteria predicting outcomes, our suggestion is to continue the current standard of care of offering surgical repair to patients who may clinically benefit from a procedure. Our suggestion against refraining from surgical intervention is derived from the pathophysiology of ATAAD and its role in malperfusion. In the setting of ischemia, there is no intuitive reason to delay reperfusion. Minimizing the duration of ischemia in other ischemic pathologies such as myocardial ischemia and cerebrovascular accidents is the priority, and without evidence to the contrary, physicians should aim to repair the affected portion of the vasculature at the earliest opportunity. Management of acute dissection is complex and requires adequate institutional experience and the ability to deploy resources with minimal notice. Malperfusion management followed by inflow correction is physiologically the best course of action to achieve an ideal clinical outcome.

The current primary literature shows that advanced age [13], shock at presentation, cardiac tamponade, myocardial infarction, along with local and systemic malperfusion, are correlated with worse outcomes post ATAAD repair [14]. Based on these risk factors, two scoring criteria have been developed. The Penn Classification [61] and German Registry of Acute Type A Dissection (GERAADA) score [11] use preoperative clinical and radiologic data to predict in-hospital and 30-day postoperative mortality. The GERAADA score considers the patients’ age, sex, previous cardiac surgery, inotropic support, resuscitation before surgery, aortic regurgitation, preoperative hemiparesis, intubation/ventilation status, preoperative organ malperfusion, extension of aortic dissection, and location of primary entry site as determined by computed tomography (CT) imaging [21]. The Penn Classification system consists of four classes: Class A is the absence of malperfusion or circulatory collapse; Class B consists of manifestations of malperfusion such as stroke, paraplegia, focal neurological deficits, acute kidney injury, and mesenteric ischemia; Class C is characterized by circulatory collapse exemplified by acute ventricular dysfunction, pericardial tamponade, acute coronary ischemia, or myocardial infarction; and Class B + C meets at least one criterion of both classes [61]. Notably, neither of these risk stratification models considers lactate in their calculations, but the effort to quantify malperfusion as a predictor for poor outcomes was established. Despite external validation, the Penn Classification is yet to be widely adopted due to its reliance on advanced imaging and intraoperative findings [5].

Currently, only a handful of studies have explored the predictive value of lactate as an independent predictor of outcomes in ATAAD. Although these studies found that increased preoperative lactate predicted worse mortality, the optimal lactate cut-off value for risk stratification widely ranged from 2.75 mmol/L [19] to 3.2 mmol/L [18] to 6.0 mmol/L [5]. As expected, when taken together, the results of these studies showed that as the cutoff value for “high lactate” increased, so did its discriminatory power. This reinforces that there is an optimum cut-off value above which surgical repair will not provide any benefit in mortality. However, due to the variety in the optimal cut-off value, a suggestion for a single numeric value to use is difficult to make. Unfortunately, the inability to parse through the raw data from each of the studies examined instead of having to rely on averages for serum lab values prevented us from calculating the optimum lactate cut-off values with a much larger sample size than previously studied. Additionally, the source of variability in optimal cut-off values included in these studies is difficult to determine but is likely due to small sample size, the nature of retrospective studies, and a lack of standardization across several institutions, which includes factors such as lactate measurement timing. Even in the setting of a low cut-off value, these data would allow further detailed investigation into the possibility of using a combination of multiple easily obtained lab values to develop criteria similar to the Penn Classification and GERADA but more time-efficient to obtain.

Predictive tools for risk stratification are one component of providing personalized medicine in the setting of high-risk, complex aortic procedures for ATAAD. Incorporating lactate as a biomarker in surgical risk assessment can enhance and individualize decision-making by providing insight into tissue perfusion at the time of presentation. Mokhles et al. emphasize that optimal surgical care finds a balance between evidence-based and personalized medicine, ensuring that individual patient values and expectations, physician skills and experience, and best available evidence are incorporated into the clinical decision-making [62]. Utilizing lactate alongside existing risk assessment tools strengthens evidence to guide surgical planning and risk assessment. Additionally, added evidence simply enhances patient counseling on surgical risks and potential outcomes. Improved communication, which includes discussion of personalized risk, can allow for a shared decision-making process that sets clear expectations of the planned surgery.

Despite the valuable information reviewed in this study, there were several limitations. Firstly, confounders of serum lactate levels were not considered, such as liver function enzymes and hemolysis. Additionally, although only acute aortic dissections were analyzed, the precise duration of symptom onset prior to presentation was not available for all participants. Naturally, the findings are biased due to the retrospective nature of this study, including selection bias of only patients included in prior publications. As previously mentioned, the inability to sieve through the raw data and only look at averages may introduce bias in the results as well. Lastly, this review did not consider long-term outcomes and postoperative morbidity associated with ATAAD, including respiratory failure, renal failure, stroke, and mesenteric ischemia, which could also affect a family’s decision to pursue surgery in the first place.

## 5. Conclusions

In conclusion, the role of lactate in assessing mortality in patients with ATAAD is undeniable and is likely larger than other easily obtained lab values such as BUN, creatinine, INR, platelets, and hemoglobin. However, due to the lack of uniformity in optimal cut-off levels for risk stratification in the current literature, the use of lactate or LDH as an independent predictor of outcome is ill-advised. Rather, lactate considered in conjunction with other clinical, laboratory, and radiographic findings is more optimal. The addition of lactate alongside additional biomarkers such as inflammatory markers (ESR and/or CRP as examples) and/or troponins can be used to frame future work. This approach would be particularly novel since personalized decisions towards repairs are more likely to be successful. Additionally, due to the lack of risk-stratification-scoring tools that include lactate, future studies should consider ways to add lactate to current scoring tools such as GERAADA or the Penn Classification or develop separate tools with its inclusion.

## Figures and Tables

**Table 1:** Combined data and results.

	Sample Size	Male	Mean Age	Mortality Rate	Pre-op Lactate (mmol/L)	LDH (U/L)	BUN (mg/dL)	Creatinine (mg/dL)	INR	PT (Second)	PTT (Second)	Platelets (10^9^/L)	Hemoglobin (g/dL)
Gemelli [18]	128	88	66	14.84%	1.7								
Benett [5]	144	96	59	9.00%	4.0			1.13					
Saritas [20]	50	17	56	2.00%		431							
Zindovic [19]	277	174	63	13.00%	2.6			1.09					
Ghoreishi [21]	269	179	59	16.00%	2.2			1.25			35.4		12.6
Kofler [22]	632	339	62	13.80%	2.3			1.10					
Luo [23]	225	181	53	13.87%	1.6			1.01				164.0	
Wu [24]	141	105	51	5.67%	1.9			1.17		15.7			12.9
Pan [25]	1154	781	63	17.62%	2.1								
Leshnower [26]	34	26	53	55.88%	4.3								
Oz [27]	57	9	55	26.30%	3.0							194.3	
Zhang [28]	60	43	55	46.67%	2.3		6.3	0.90				169.9	13.0
Wang [29]	503	370	54	14.51%	3.5		8.9	0.88	1.2	13.1		145.4	12.1
Hou [30]	27	22	53	81.48%	1.3		8.0	1.49				195.0	13.1
Yamashiro [31]	10	4	56	20.00%	3.7								
Slaven [32]	98	63	64	34.70%	2.0			1.12					13.8
Jin [33]	121	93	47	6.61%	1.3							180.0	12.7
Gong [34]	74	54	48	12.16%	1.6			0.98					
Suliman [35]	1	1	63	0%	11.9								
Yang [36]	40	25	54	10.00%	2.3			1.14					
Kamenskaya [37]	58	45	53	12.07%	0.95								
Hiraya [38]	1	1	64	0%	3.8								
Jin [39]	100	72	47	9.00%	1.4							177.0	12.7
Guné [40]	336	224	64	13.69%	1.7			0.99					
Mariscalco [41]	62	45	63	74.20%	6.8								
Uehara [42]	34	19	69	61.76%	10.1								
Li [43]	42	40	49	4.80%	5.4			1.26					
Rublee [44]	1	1	59	0%	16.6								
Meriggi [45]	1	1	38	0%	16.0			1.50					
Tolefac [46]	1	1	53	100%		455		1.72					15.2
Neira [47]	1	1	67	0%	6.3			1.19	1.2		30.0	174.0	13.7
Robu [48]	1	1	51	0%	2.3			2.59	1.4				
Fujiyoshi [49]	1	1	48	0%		213							
Kusadokoro [50]	1	1	60	0%	7.5								
Mufty [51]	1	1	53	100%	4.1								
Murali [52]	1	0	71	0%	12.2		14	0.41					5.6
Memon [53]	1	0	45	0%	1.1								
Lazar [54]	1	1	54	0%	1.1		20	1.00					
Nguyen [55]	1	0	31	0%	7.2			3.02					
Jelani [56]	1	1	56	0%	4.7			5.54					
Hisata [57]	1	1	64	0%	11.1	314							
Muzzi [58]	1	1	62	0%	12.0								
Aoyama [59]	1	0	83	0%	1.7								
Johnston [60]	1	0	34	0%	3.0								
Totals	4695	66.6%	59.2	16.8%	2.4	425	8.6	1.06	1.2	13.7	35.4	161.1	12.6

## Data Availability

No new data were created or analyzed in this study. Data sharing is not applicable to this article.

## Abbreviations

The following abbreviations are used in this manuscript:

ATAAD	Acute Type A aortic dissection
LDH	Lactate dehydrogenase
GERAADA	German Registry of Acute Type A Dissection
BUN	Blood urea nitrogen
INR	International normalized ratio
PT	Prothrombin time
PTT	Partial thromboplastin time
